# Corrected and Republished from: A Nonfunctional Opsonic Antibody Response Frequently Occurs after Pneumococcal Pneumonia and Is Associated with Invasive Disease

**DOI:** 10.1128/mSphere.01102-20

**Published:** 2020-12-16

**Authors:** Fabian Uddén, Jonas Ahl, Nils Littorin, Kristoffer Strålin, Simon Athlin, Kristian Riesbeck

**Affiliations:** a Clinical Microbiology, Department of Translational Medicine, Faculty of Medicine, Lund University, Malmö, Sweden; b Infectious Diseases, Department of Translational Medicine, Faculty of Medicine, Lund University, Malmö, Sweden; c Unit of Infectious Diseases, Department of Medicine Huddinge, Karolinska Institutet, Stockholm, Sweden; d Department of Infectious Diseases, Faculty of Medicine and Health, Örebro University, Örebro, Sweden; U.S. Food and Drug Administration

**Keywords:** adaptive immune response, bacteremia, immunoglobulins, opsonization, phagocytosis, pneumonia, *Streptococcus pneumoniae*, adaptive immunity

## Abstract

Naturally acquired opsonic antipneumococcal antibodies are commonly found in nonvaccinated adults and confer protection against infection and colonization. Despite this, only limited data exist regarding the adaptive immune response after pneumococcal exposure. To investigate the dynamics of naturally acquired antipneumococcal immunity in relation to an episode of infection, opsonic antibody activity was studied with paired acute-phase and convalescent-phase sera obtained from 54 patients with pneumococcal community-acquired pneumonia (CAP) using an opsonophagocytic assay (OPA). Results were compared with clinical characteristics and anticapsular immunoglobulin (Ig) concentrations. Interestingly, a nonfunctional opsonic antibody response (characterized by a decreased convalescent-phase serum OPA titer compared to that of the acute-phase serum or undetectable titers in both sera) was observed in 19 (35%) patients. The remaining individuals exhibited either an increased convalescent-phase OPA titer (*n * = * *24 [44%]) or a detectable, but unchanged, titer at both time points (*n *=* *11 [20%]). Invasive pneumococcal disease (i.e., bacteremia) was significantly more common among patients with a nonfunctional convalescent-phase response than in patients with other convalescent-phase responses. Anticapsular Ig concentrations were higher among patients with detectable convalescent-phase OPA titers (*P* = 0.003), and the greatest Ig concentration increase was observed among patients with an increased convalescent-phase response (*P* = 0.002). Our findings indicate that an episode of pneumococcal infection may act as an immunizing event. However, in some cases when patients with CAP also suffer from bacteremia, a nonfunctional opsonic antibody response may occur. Furthermore, the results suggest that factors other than anticapsular Ig concentrations determine opsonic antibody activity in serum.

**IMPORTANCE** Numerous reports on the dynamics of antipneumococcal immunity in relation to immunization with pneumococcal vaccines and on the prevalence of naturally acquired immunity in various populations have been published. In contrast, studies on the dynamics of the humoral immune response triggered by pneumococcal infection are scarce. This study provides valuable information that will contribute to fill this knowledge gap. Our main results indicate that a functional immune response may fail after CAP, predominantly among patients with simultaneous bacteremia.

## Abstract (precis)

Numerous reports on the dynamics of antipneumococcal immunity in relation to immunization with pneumococcal vaccines and on the prevalence of naturally acquired immunity in various populations have been published. In contrast, studies on the dynamics of the humoral immune response triggered by pneumococcal infection are scarce.

## INTRODUCTION

Streptococcus pneumoniae is a human respiratory tract pathogen responsible for substantial morbidity and mortality on a global scale, causing community-acquired pneumonia (CAP), acute otitis media, and rhinosinusitis as well as invasive pneumococcal disease (IPD) (1, 2). The capsular polysaccharide (CPS), which mediates protection from phagocytosis, is the most important virulence factor of S. pneumoniae. CPSs of various chemical compositions may induce the production of specific immunoglobulins (Igs) upon exposure, defining pneumococcal serogroups and serotypes. Serotype-specific anti-CPS Igs that mediate opsonophagocytosis are generally regarded as the most important factor for immunologic protection against pneumococcal infection, and purified CPSs from many serotypes have therefore been used in pneumococcal vaccine formulae for more than half a century (3, 4).

Naturally acquired pneumococcal-antibody-mediated immunity is prevalent in young adults and confers protection against colonization and infection but declines with age (5, 6). Episodes of asymptomatic nasopharyngeal colonization induce protective adaptive immunity, but studies on the dynamics of naturally acquired immunity related to episodes of clinically significant pneumococcal infection are scarce (7). Interestingly, a delayed or absent anti-CPS Ig increase has been described after pneumococcal bacteremia (8–10). This observation raises the question whether infection by pneumococci, unlike an episode of asymptomatic colonization, may fail to induce immunologic boosting. It should, however, be noted that naturally acquired pneumococcal immunity is dependent on antibodies directed against protein antigens and cell-mediated immunity in addition to anti-CPS Ig (11, 12). Thus, the functionality of this response may not be fully evaluated solely by the measurement of anti-CPS Ig concentrations, as has been done in the above-mentioned studies.

Although pneumococcal vaccination has been highly successful in reducing morbidity and mortality, hyporesponsiveness may occur after vaccination in certain clinical situations (13–16). Suboptimal vaccine responses, as well as waning naturally acquired immunity, are linked to reduced opsonic Ig function and diminished B-cell populations (5, 17–21). Consequently, studies on the adaptive immune response after pneumococcal infection are important for increased understanding of a mechanism(s) that may impact vaccine-induced immunity.

In the current study, we investigated the correlation between opsonic antibody activity in serum against the infecting pneumococcal serotype, as measured by an opsonophagocytic assay (OPA), and disease severity as well as other clinical factors in patients with pneumococcal CAP. Serum samples were obtained from a cohort of CAP patients with previously measured total Ig concentrations against CPS of the infecting serotype and pneumococcal DNA load in plasma (22). Interestingly, lower levels of anti-CPS Ig were observed in acute-phase sera from bacteremic patients than nonbacteremic patients, and any distinct Ig increase in convalescent-phase sera did not occur in more than half of the cases in that study. By analyzing these samples with a functional method such as OPA, which may be affected also by Ig directed to non-CPS targets, and by testing the functionality rather than the quantity of antibodies (3, 12), we aimed to further improve the understanding of the naturally occurring immune response to pneumococcal infection.

## RESULTS

Available acute-phase and convalescent-phase sera from patients (*n *=* *54) infected with a serotype included in the 13-valent pneumococcal conjugate vaccine (PCV13) were obtained from a cohort of patients with radiologically confirmed CAP (22). Demographic and clinical characteristics of the individuals studied are presented in Table 1, and individual data used in analyses are outlined in Table S1 in the supplemental material.

**TABLE 1:** Patient characteristics and their relation to outcome in an OPA^*a*^

Patient characteristic	Values for:							
	All patients	Patients with an acute-phase serum OPA titer of:		*P*	Patients with a convalescent-phase serum OPA titer that was:			*P*
		>1	1		Increased	Unchanged	Decreased or undetectable	
No. (%)	54 (100)	28 (52)	26 (48)		24 (44)	11 (20)	19 (35)	
Age [median no. of yrs (range)]	69 (23–91)	75 (23–91)	60 (31–89)	**0.032**	61 (31–90)	78 (46–89)	69 (23–91)	**0.028**
Age >65 yr [no. (%)]	29 (54)	19 (68)	10 (39)	**0.030**	10 (42)	9 (82)	10 (53)	0.086
Female [no. (%)]	25 (46)	13 (46)	12 (46)	0.98	10 (42)	4 (36)	11 (58)	0.43
Current smoking [no. (%)]	16 (30)	8 (29)	8 (31)	0.86	7 (29)	2 (18)	7 (37)	0.56
Comorbidity^*b*^ [no. (%)]	26 (48)	14 (50)	12 (46)	0.78	10 (42)	8 (73)	8 (42)	0.19
CRB-65 [median (range)]	1 (0–4)	1 (0–4)	1 (0–3)	0.41	1 (0–4)	1 (0–3)	1 (0–3)	0.86
Bacteremia [no. (%)]	16 (30)	6 (21)	10 (39)	0.17	5 (21)	1 (9)	10 (53)	**0.019**
Sepsis^*c*^ [no. (%)]	34 (63)	19 (68)	15 (58)	0.44	14 (58)	7 (64)	13 (68)	0.79
SOFA score increase [median (range)]	2 (0–5)	2 (0–5)	2 (0–4)	0.17	2 (0–5)	2 (1–4)	2 (0–4)	0.81
Symptom duration^*d*^ [median no. of days (range)]	3 (0–36)	4 (0–36)	3 (0–11)	0.51	3 (0–21)	1 (0–36)	5 (0–11)	0.078
CRP maximum [median (range)]	298 (42–773)	262 (42–611)	328 (44–773)	0.44	342 (62–611)	171 (42–495)	315 (44–773)	0.050
No. of day between sample collections [median (range)]	31 (20–82)	31 (20–81)	29 (24–82)	0.27	31 (25–81)	33 (25–69)	29 (20–82)	0.29

a Characteristics of 54 patients with pneumococcal CAP and results in an opsonophagocytic assay (OPA) for corresponding paired acute-phase and convalescent-phase sera. Patients are grouped according to a detectable (>1) or undetectable (1) acute-phase serum OPA titer, as well as the change of the OPA titer from the acute phase to convalescence. Associations of these outcomes with individual patient characteristics were analyzed. Statistically significant differences between groups (*P* < 0.05) are indicated in bold type. CRB-65, confusion of new onset, respiratory rate of 30 breaths/min, systolic blood pressure of <90 mm Hg or diastolic blood pressure of 60 mm Hg or less, age of 65 years or older; SOFA, sequential organ failure assessment; CRP, C-reactive protein.

b One or more of any of the following diagnoses: chronic obstructive pulmonary disease (COPD), heart disease, diabetes mellitus, liver disease, renal insufficiency, neoplasm, or immunosuppression.

c Fulfillment of the Sepsis-3 definition (33).

d Number of days with symptoms of pneumonia before collection of acute-phase serum.

10.1128/mSphere.01102-20.1 TABLE S1 List of included patients, demographic and clinical data, serotype-specific anti-CPS Ig concentrations, and pneumococcal DNA load in plasma. The concentration of pneumococcal Spn9802 DNA in plasma was available for 25 of the included patients from whom EDTA plasma samples had been collected during the acute phase of infection. Download Table S1, DOCX file, 0.03 MB. Copyright © 2020 Uddén et al. 2020 Uddén et al. This content is distributed under the terms of the Creative Commons Attribution 4.0 International license.

### A nonfunctional opsonic antibody response is observed in approximately one-third of patients after CAP.

**FIG 1:**
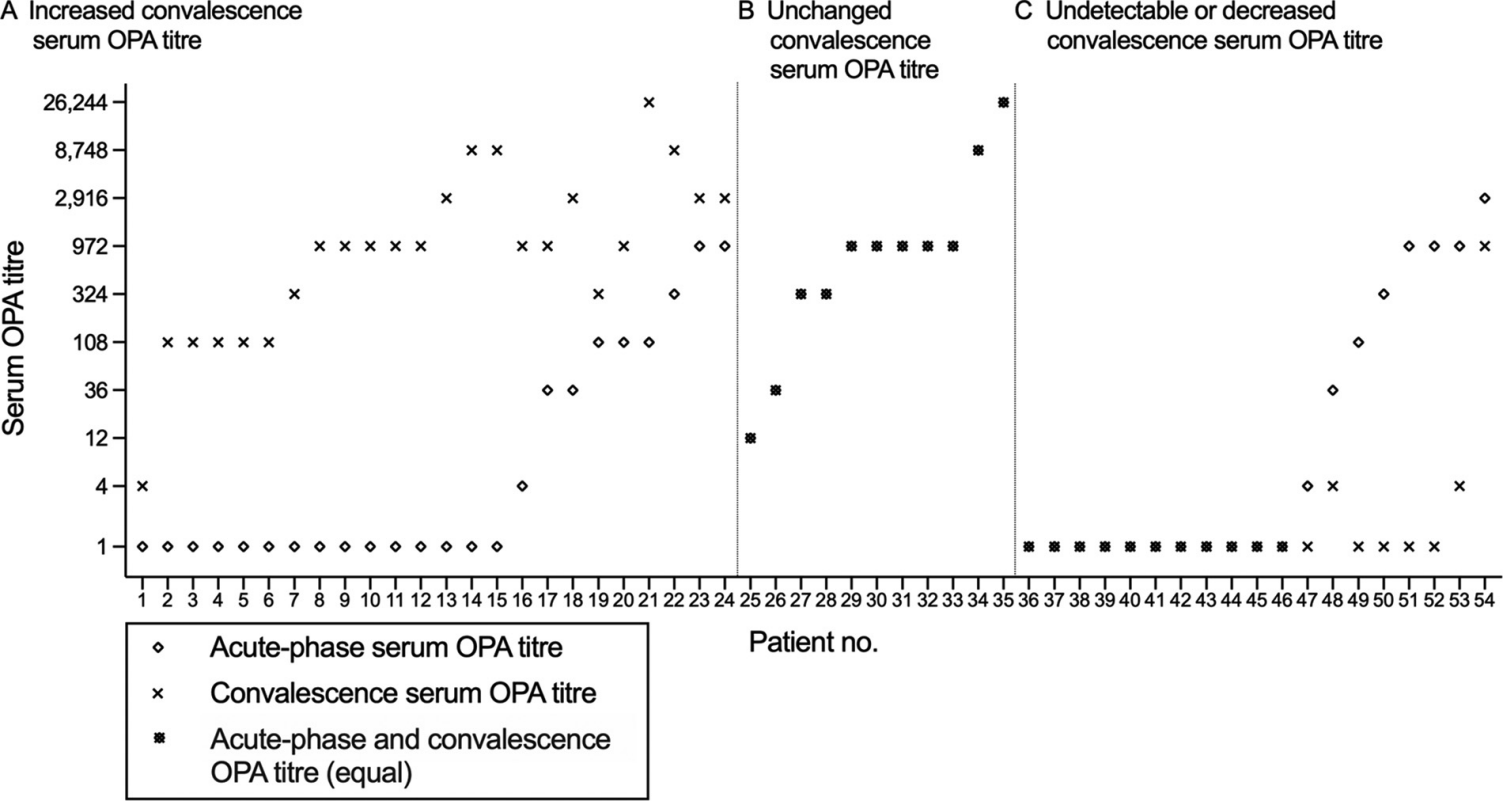
Acute-phase and convalescent-phase opsonic antibody activities in sera from patients with pneumococcal pneumonia. Opsonophagocytic assay (OPA) titers in paired sera during the acute phase and convalescence of 54 patients with pneumococcal community-acquired pneumonia were obtained. Detectable OPA titers (i.e., an OPA titer of >1) were found with sera from 28 (52%) and 38 (71%) patients during their acute phase and convalescence, respectively. The remaining samples did not induce any bacterial killing in the presence of complement and phagocytes (i.e., the OPA titer was 1). (A) Convalescent-phase sera from almost half of the studied patients (*n *=* *24 [44%]) yielded increased OPA titers compared to titers in the corresponding acute-phase sera. (B) With sera from 11 patients (20%), equal OPA titers were observed at both sampling times. (C) Finally, OPA titers were undetectable (i.e., their OPA titer was 1) at both sampling times with sera from 11 patients (20%), and decreased OPA titers were yielded with convalescent-phase sera from 8 patients (15%) compared to corresponding acute-phase sera. These patterns were assessed as nonfunctional opsonic antibody responses (35%).

To investigate the adaptive immune response in patients with CAP, an OPA was performed on acute-phase and convalescent-phase sera by measuring opsonic antibody activity against the infecting pneumococcal serotype. OPA titers were calculated based upon the dilution of serum resulting in >50% bacterial killing in the presence of complement and phagocytic cells compared to the level of bacteria in negative controls. If no bacterial killing was observed (i.e., the serum OPA titer was undetectable), the tested serum was assigned an OPA titer of 1. The OPA titers yielded, with corresponding sera, from each individual patient are visualized in Fig. 1. Three divergent convalescent-phase opsonic antibody responses were observed when paired sera were compared; the convalescent-phase serum OPA titer was either increased (44%) (Fig. 1A), unchanged (20%) (Fig. 1B), or undetectable/decreased (35%) (Fig. 1C) compared to the acute-phase serum OPA titer. Patients with decreased or undetectable convalescent-phase serum OPA titers were defined as exhibiting a nonfunctional convalescent-phase opsonic antibody response.

To exclude Ig deficiencies that may explain a low opsonic activity, IgG and IgG2 concentrations were measured in all acute-phase sera, and lower reference limits defined in clinical guidelines (6,700 μg/ml and 1,150 μg/ml, respectively) were used for evaluation. No significant IgG or IgG2 deficiencies were detected in any of the patient sera tested.

### Bacteremia is associated with a nonfunctional convalescent-phase opsonic antibody response.

To explore whether the opsonic antibody function was associated with any clinical or demographic features, results from OPA were correlated with various factors as outlined in Table 1. In these analyses, we compared patients with undetectable or detectable acute-phase serum OPA titers (i.e., an OPA titer of 1 or >1, respectively) and patients with differing convalescent-phase responses (Fig. 1). During the acute phase, undetectable OPA titers were significantly more common among patients younger than 65 years of age (68% versus 39%; *P* = 0.032) than among older patients. However, the lowest median age (61 years) was found in those with an increased convalescent-phase OPA titer (*P* = 0.028). To further investigate any association of opsonic antibody response with age, outcomes in the OPA were compared between stratified age groups (Table 2). No statistically significant differences could be observed using these small groups, but it was noted that 7/10 patients aged <50 years developed an increased convalescent-phase response, whereas only 2/13 patients among those aged ≥80 years developed an increased response.

**TABLE 2:** Outcome in OPA stratified by age group

Age group (yr)	No. of patients	No. (%) of individuals with:				
		An acute-phase serum OPA titer of:		A convalescent-phase serum OPA titer that was:		
		>1	1	Increased	Unchanged	Decreased or undetectable
<50	10	5 (50)	5 (50)	7 (70)	1 (10)	2 (20)
50−59	10	2 (20)	8 (80)	4 (40)	1 (10)	5 (50)
60−69	8	4 (50)	4 (50)	5 (63)	0	3 (38)
70−79	13	7 (54)	6 (46)	6 (46)	4 (31)	3 (23)
≥80	13	10 (77)	3 (23)	2 (15)	5 (38)	6 (46)

*P*		0.12		0.083^*a*^		

a The chi-square test was performed with dichotomous outcome variables by pooling the “unchanged” and “decreased or undetectable” groups.

Importantly, bacteremia was more common among patients with a nonfunctional convalescent-phase opsonic antibody response (53%) (Fig. 1C) than among those with either an increased (21%) (Fig. 1A) or unchanged (9%) (Fig. 1B) convalescent-phase OPA titer (*P* = 0.019), suggesting an association between IPD and an attenuated immune response against pneumococci after infection.

### OPA titers and anticapsular Ig concentrations correlate during the convalescent phase but not the acute phase.

**FIG 2:**
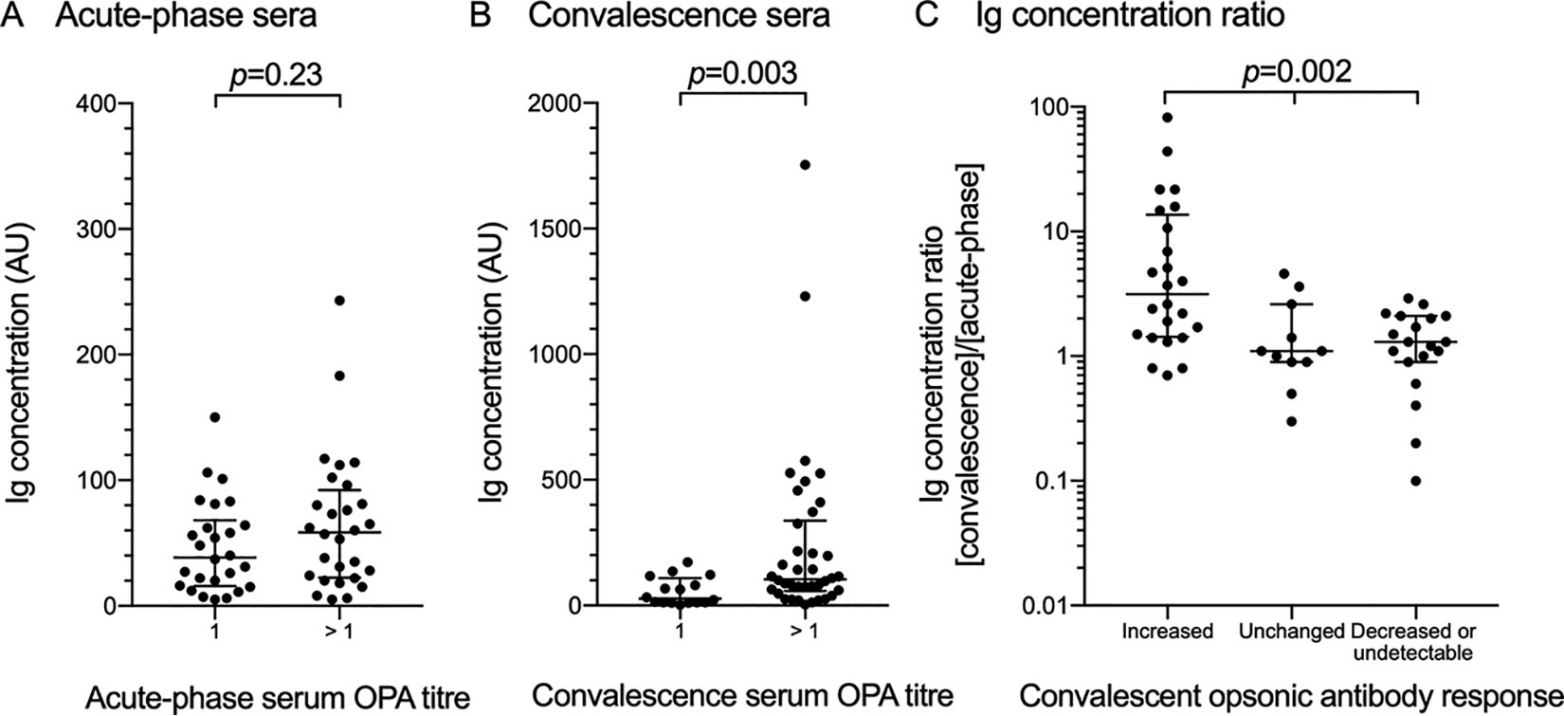
Antipneumococcal Ig concentrations compared to OPA titers and convalescent-phase opsonic antibody responses. Serotype-specific anti-capsular polysaccharide (anti-CPS) total Ig concentrations (in arbitrary units [AU]) (measured by ELISA) in sera from 54 patients with pneumococcal community-acquired pneumonia in relation to corresponding serum OPA titers. Ig concentrations and OPA titers were measured during both the acute phase of infection (A) and during convalescence (B). (C) The Ig concentration fold change from acute phase to convalescence (convalescence/acute-phase Ig concentration ratio) in serum from each patient was compared with their opsonic antibody response. Error bars indicate the interquartile range of median Ig concentration and median Ig concentration ratio.

To determine whether serotype-specific anti-CPS Ig concentrations may explain the different titers observed in the OPA, total Igs reacting with CPSs were measured by an enzyme-linked immunosorbent assay (ELISA) (22). Acute-phase and convalescent-phase sera from all patients are presented in Fig. 2 and related to the results in the OPA. During the convalescent phase, the median Ig level was higher among individuals with detectable OPA titers compared to those with undetectable OPA titers (105 arbitrary units [AU] versus 28 AU; *P* = 0.003), while no difference was observed during the acute phase (Fig. 2A and B). In parallel, convalescent/acute-phase Ig concentration ratios (Ig fold change) differed between the groups with diverging convalescent-phase responses, with the greatest median Ig fold change observed among patients with an increased convalescent-phase OPA titer (*P* = 0.002) (Fig. 2C). Albeit the above associations between outcome in OPA and Ig levels/ratios were observed, low Ig levels were also found with patient sera exhibiting high OPA titers and vice versa. These results indicate that factors other than anticapsular Ig may have influenced opsonic activity of the studied patient sera.

## DISCUSSION

In the present study, we used a single-serotype OPA to study the antibody-mediated opsonic activity against pneumococci in sera from adult patients with pneumococcal CAP. By examining paired acute-phase and convalescent-phase sera, the dynamics of the immune response could be presented and assessed. Almost half of the patients (44%) responded with a functional immune response characterized by an improved opsonic antibody function in sera collected approximately 1 to 3 months after the acute phase of infection. Conversely, we found that a surprisingly large proportion of the patients (35%) failed to develop detectable OPA titers or exhibited decreased opsonic activity in serum during convalescence, a response that was considered nonfunctional. The remaining patients (20%) exhibited detectable, but unchanged, OPA titers at both sampling times, a pattern that is not easily interpreted as functional or nonfunctional. Invasive pneumococcal disease was clearly associated with a nonfunctional opsonic antibody response. Most importantly, our observations reveal that an episode of clinically significant pneumococcal infection often results in immunization and induces an improved humoral immune status against the infecting serotype; however, a nonfunctional opsonic antibody response may occur in some cases.

It is an interesting observation that 28 individuals developed pneumococcal CAP despite exhibiting functional serum opsonic antibody activity during the acute phase, 6 of whom had bacteremia. Protection against mucosal pneumococcal infection is, however, dependent on a well-functioning innate and humoral immunity (11); the latter was not assessed in the current study. Although it is a well-known fact that opsonic antibodies are important for preventing IPD (6, 7, 23), we did not find a significant association between undetectable acute-phase serum OPA titers and bloodstream infection in the current study. This may be due to the use of a low cutoff (i.e., an OPA titer of >1), as an OPA titer as high as 64 has previously been suggested to correlate with protective immunity in adults (3). However, we chose this cutoff to avoid arbitrary grouping of the patients, as more studies are needed to clearly establish a relationship between specific serum OPA titers and protection from pneumococcal infection in adults (3).

Few previous studies on the dynamics of naturally acquired pneumococcal immunity related to an infection episode exist. An association of pneumococcal antigenemia with an attenuated convalescent-phase quantitative anti-CPS Ig response was, however, reported in 1976 (8), and decreased serotype-specific Ig concentrations following pneumococcal infection have been described in case reports (9, 10). In one previous study (24), 88% of convalescent-phase sera from patients with nonbacteremic pneumonia had functional opsonic Ig compared to 50% of sera from bacteremic patients. Similarly, we observed that 82% of nonbacteremic patients and 44% of bacteremic patients had detectable OPA titers in their convalescent-phase sera. These findings suggest that impaired opsonic activity against the homologous serotype may be common subsequent to IPD, but the precise mechanism(s) to this reaction is at present unknown.

Naturally acquired immunity to pneumococci during a life span is characterized by decreasing anti-CPS Ig levels as well as opsonic Ig function with increasing age, explained by infrequent immune boosting due to exposure to the bacterium and immunosenescence (5, 6, 25). Pneumococcal carriage rates among adults are low in northern Europe, thus possibly contributing to infrequent pneumococcal exposure and decreased immunity against pneumococci (26). However, we observed an association between age above 65 years and detectable acute-phase OPA titers, which may contradict the results of these previous studies. On the other hand, the patients who responded with a functional immune response were generally younger, which further supports that high age is linked to impaired immunity against S. pneumoniae.

An inability to mount an appropriate antibody response, hyporesponsiveness, has also been seen in studies on pneumococcal vaccines (13–16, 18). This is thought to be associated with an exhausted memory B-cell pool due to high levels of circulating CPS, either at the time of immunization or during previous exposure. Since the T-cell-independent response induced by pure polysaccharide antigens does not result in memory B-cell propagation, adaptive immunity may be reduced after repeated exposures (19–21). Likewise, the impaired immune response observed in the current study might also have been influenced by exposure to a high level of circulating CPS, either during previous colonization or infection or during the studied CAP episode. This is in line with previous studies that suggest an association between antigenemia and a poor quantitative Ig response (8, 10). Moreover, we found that the two patients with the highest pneumococcal DNA concentration in plasma (see Table S1 in the supplemental material) exhibited nonfunctional convalescent-phase responses. This observation further supports the hypothesis that systemic dissemination during infection may prevent a positive adaptive immune response. However, impaired immune responses were also observed in patients with low levels of circulating Spn9802 DNA.

We recently investigated the association between OPA titers and different serotypes in 40 patients with IPD, including 14 of the currently studied patients, and found that serotypes with a thick CPS layer were more prone to cause an impaired convalescent-phase opsonic response (27). This may be due to a high concentration of CPS in the bloodstream caused by serotypes producing a thick capsule and resulting in a hampered convalescent-phase B-cell response. In the current study, the proportions of these infecting serotypes did not differ between bacteremic and nonbacteremic patients (data not shown), and no measurement of free CPS in the bloodstream was available, which is why we cannot conclude whether and to what extent this mechanism may explain observed differences.

We found that serotype-specific Ig concentrations varied considerably at both time points, but a statistically significant correlation with corresponding serum OPA titers was detected during the convalescent phase (Fig. 2). Anti-CPS Ig concentrations and OPA titers in preimmunization sera from adults have previously been reported to weakly correlate but may be improved after pneumococcal vaccination (3, 6, 24, 28). Our results indicate that a similarly improved correlation is induced by an episode of pneumococcal infection. In studies of both naturally acquired and vaccine-induced antibodies, the discrepancy between Ig concentration and OPA titers has been shown to be partly due to IgG with low avidity, a factor that may have contributed to the current results (24, 29, 30). Various concentrations of Ig directed against pneumococcal proteins may contribute to the effectiveness of opsonophagocytosis and might also explain inconsistencies between OPA titers and anti-CPS Ig levels (12). Even if the results of an OPA better reflect the function of antibody-mediated immunity than purely quantitative assays, it is important to mention that nonopsonic anticapsular antibodies (that do not affect readouts in OPA) have been found to prevent pneumococcal colonization as well as dissemination in mice (31). Consequently, it should be kept in mind that functional assays based on opsonophagocytosis do not constitute a perfect surrogate marker for antipneumococcal humoral immune status.

There are a few limitations of this study. First, due to the small number of patients included, the study should be regarded as exploratory; larger studies are needed to confirm our results. Second, based on a low prevalence of asymptomatic pneumococcal carriage in adults in Northern Europe, we regarded a pneumococcal serotype as possible true etiology if it was detected in cultures from the nasopharynx as well as from sputum, in accordance to Swedish clinical guidelines (26, 32). It is, however, possible that CAP was caused by a different etiological agent in patients with positive culture only from the nasopharynx. Furthermore, no information on previous exposure to pneumococci or immunization with the 23-valent pneumococcal polysaccharide vaccine was available for the studied individuals, but prescription rates of this vaccine have been low (1 to 2 doses per 10,000 adults per year) during the last 2 decades (https://sdb.socialstyrelsen.se/if_lak/val.aspx), and it was administered only to certain risk groups during the study period, indicating a low probability that any of the study subjects had received it. Finally, it should be noted that the lack of mass quantity assignments in the ELISA results for Ig concentrations, from which results were provided in arbitrary units (AU), is a limitation. However, the use of AU for comparison of serum Ig concentrations between different patients have been assessed as sufficient for the objectives of the current study.

In conclusion, despite the fact that infection with pneumococci resulted in an improved humoral immune response during convalescence in almost half of the patients in our cohort, approximately one-third of patients did not develop functional opsonic antibodies and even exhibited a decreased immune function in some cases, a response that was significantly associated with IPD. The high systemic CPS load and high age might possibly contribute to the failed immune response observed.

## MATERIALS and METHODS

### Study population.

Study patients were part of a cohort at Örebro University Hospital consisting of 235 adults with CAP admitted to the Department of Infectious Diseases. Inclusion criteria and group characteristics of the original cohort have been described previously (22). CAP was radiologically verified and defined as acute onset of illness with two of the following signs or symptoms: fever of ≥38°C, dyspnea, cough, pleuritic chest pain, and abnormal lung auscultation. Serum and plasma samples were collected from the patients within 2 days of admission (acute phase) and after approximately 1 to 3 months (convalescence). The duration from onset of illness until hospital admission was based on the history of the patient and was collected from the medical records. To estimate disease severity at admission, the sequential organ failure assessment (SOFA) score was calculated. Patients with an increase in SOFA score of ≥2 from baseline levels were regarded as having sepsis, in accordance with the Sepsis-3 definition (33).

Blood samples and samples from sputum and the nasopharynx were collected at admission. A Bactec blood culturing system (Becton, Dickinson, Sparks, MD) was used for blood culture. The sputum and nasopharyngeal specimens were cultured according to standard microbiological methods. All isolates of S. pneumoniae from blood cultures and cultures from respiratory tract secretions were stored at −70°C and transported in a frozen state to the Statens Serum Institut in Copenhagen, Denmark, for serotyping by the Quellung reaction (34). Bacteremic CAP was defined as growth of pneumococci in blood culture, whereas nonbacteremic CAP was defined as pneumococci cultured only from sputum or nasopharyngeal secretions.

We included patients (*n *=* *54) from a previous cohort (22) who were infected by a serotype included in PCV13 and from whom paired sera (acute-phase and convalescent-phase sera) were available. The median age was 68.5 years (range, 23 to 91 years). Twenty-five patients were female (46%). Pneumococci grew in blood cultures from 16 (30%) patients, whereas 38 (70%) had nonbacteremic CAP. Among the patients with nonbacteremic CAP, pneumococci were isolated in sputa from 23 patients, whereas the nasopharynx was the only site of isolation for 15 patients. The median time period between paired sera was 30.5 days (range, 20 to 82 days). Infecting serotypes were 3 (*n *=* *11), 14 (*n *=* *10), 7F (*n *=* *9), 23F (*n *=* *6), 9V (*n *=* *4), 18C (*n *=* *3), 19A (*n *=* *3), 19F (*n *=* *3), 1 (*n *=* *2), 4 (*n *=* *2), and 6B (*n *=* *1). Sera (*n *=* *13) from patients with bacteremic CAP included in the present study were recently used in a study on the post-IPD immune response related to pneumococcal serotypes as part of a larger group (27).

### Opsonophagocytic assay.

A single-serotype opsonophagocytic assay (OPA) based on a Centers for Disease Control and Prevention protocol developed by Romero-Steiner et al. (35) available from the World Health Organization (WHO) Bacterial Respiratory Tract Pathogen Reference Laboratory (University of Alabama [UAB], Birmingham, AL; https://www.vaccine.uab.edu/uploads/mdocs/cdc-ops3.pdf) was performed on all acute-phase and convalescent-phase sera. Some modifications of the method were made according to the more recent UAB multiplexed-OPA protocol (36). Briefly, the S. pneumoniae target strain of the infecting serotype (BEI Resources, Manassas, VA) suspended in opsonization buffer B (OBB; Hanks’ balanced salt solution with Mg^2+^ and Ca^2+^ supplemented with 0.1% gelatin and 10% heat-inactivated fetal bovine serum) was added to a threefold dilution series (starting at 1:4 dilution of total assay volume) of heat-inactivated patient sera in OBB in duplicates and incubated for 30 min at room temperature (RT) to allow Ig binding to bacteria. Thereafter, samples were incubated with promyelocytic human leukemia (HL-60) cells, differentiated by propagation in 0.8% dimethylformamide for 5 days, suspended in OBB and baby rabbit complement for 45 min at 37°C in 5% CO_2_ to facilitate phagocytosis. Finally, phagocytosis was stopped by cooling samples on ice for 20 min, followed by the transfer of samples to blood agar plates and overnight culture at 37°C in 5% CO_2_. The number of CFU for each plate was manually counted. The OPA titer of a sample was defined as the inverse ratio for the weakest serum dilution titer that caused >50% killing of bacteria compared to the level of bacteria in a negative control without any serum (i.e., the remaining bacteria, ≤50% CFU). If a sample did not result in >50% killing at any concentration (i.e., the OPA titer was undetectable), it was assigned an opsonic titer of 1 for purpose of analysis and presentation. Acute-phase and convalescent-phase sera from the same individual were tested on the same microtiter plate. Patients whose convalescent-phase serum OPA titer was either undetectable or decreased compared to that of the corresponding acute-phase serum were considered to have a nonfunctional antibody response. A positive-control serum from a PCV13-immunized volunteer was run in the OPA in parallel with sera from each individual and was included in all rounds to ensure the validity of the assay. Some variability of the positive-control serum OPA titer was observed between runs, even with specimens of the same serotype, which prevented direct comparisons of OPA titers between patients (see Table S2 in the supplemental material). Regardless of this fact, the acute phase-to-convalescence dynamic of the serum OPA titer could be assessed as increased, unchanged, or decreased.

10.1128/mSphere.01102-20.2 TABLE S2 Positive-control and patient serum OPA titers listed by serotype. The paired acute-phase and convalescence sera from each included individual were examined by OPA on the same microtiter plate simultaneously as a positive-control serum. Download Table S2, DOCX file, 0.02 MB. Copyright © 2020 Uddén et al. 2020 Uddén et al. This content is distributed under the terms of the Creative Commons Attribution 4.0 International license.

### Screening for total IgG or IgG2 deficiencies and determination of antipneumococcal Ig.

A sandwich enzyme-linked immunosorbent assay (ELISA) for total IgG and IgG2 was performed on all acute-phase sera to exclude Ig deficiencies that may hamper opsonic function of sera as previously described (27). Briefly, MaxiSorp plates (Nunc, Waltham, MA) were coated with rabbit anti-human IgG antibodies (Sigma, Darmstadt, Germany) or mouse anti-human IgG2 antibodies (Sigma) overnight at 4°C. Wells were washed with wash buffer (phosphate-buffered saline [PBS] [pH 7.4], 0.05% Tween 20), followed by incubation with blocking buffer (PBS [pH 7.4], 1% skim milk, 0.05% Tween 20) for 1 h at RT. Thereafter, 10-fold dilution series of patient sera and calibration sera for IgG (Dako, Glostrup, Denmark) or IgG2 (The Binding Site, San Diego, CA) were added to plates in blocking buffer, and the plates were incubated for 1 h at RT. Following an additional wash, horseradish peroxidase (HRP)-conjugated rabbit anti-human IgG antibodies (Dako) were added to all wells, and the plates were incubated for 20 min at RT to allow binding to patient and calibration IgG or IgG2. Finally, optical density was measured at 450 nm, and absorbances of patient sera were compared to those of the calibration sera.

Antipneumococcal CPS total Ig concentrations were determined using a cell wall polysaccharide (CWPS) adsorption ELISA described by Konradsen et al. as recommended by WHO (22, 37). Briefly, sera were adsorped with CWPS before being added to MaxiSorp microtiter plates (Nunc, Roskilde, Denmark) coated with serotype-specific CPS. Ig binding was compared to that of a standard serum to calculate concentrations in AU.

### qPCR for pneumococcal DNA in plasma.

Results from examination of acute-phase plasma samples with a quantitative PCR (qPCR) for S. pneumoniae-specific Spn9802 DNA were available for 25 of the study patients. Data from these experiments were used to investigate any association with outcome in the OPA. DNA was extracted from plasma samples using an automatic NucliSENS easyMAG instrument (bioMérieux, Marcy-l'Étoile, France). After that, qPCR was used to examine the purified samples for Spn9802 DNA as previously described (38). These results have been published previously (22).

### Statistical analyses.

All statistical analyses were performed in SPSS v24 (IBM, Armonk, NY). Results were compared between any two groups using the Mann-Whitney U test and between any three groups using the Kruskal-Wallis H test. To test for equality of proportions between groups, the Pearson chi-square test or, if any cells had an expected count less than five, Fisher’s exact test was used. Differences were considered statistically significant if two-tailed *P* values were <0.05.

### Ethical approval and consent to participate.

The study was approved by the Regional Ethics Board at Lund University Hospital (approval 2012/86) and the Örebro County Council ethical committee (approval 868-1999). All patients provided their informed consent to participate in the study. The study was done in accordance with the Helsinki declaration.

### Data availability.

The data sets used and/or analyzed during the current study are available from the corresponding author on request.
