# Supplementary material for: Corrected and Republished from: A Nonfunctional Opsonic Antibody Response Frequently Occurs after Pneumococcal Pneumonia and Is Associated with Invasive Disease
Source: mSphere. 2020 Dec 16;5(6):e01102-20. doi: 10.1128/mSphere.01102-20 (PMC7758726; doi:10.1128/mSphere.01102-20)
Supplement: TABLE S1 [file mSphere.01102-20-st001.docx]

| **Table S1.** | | | | | | | | | | | | | | |
| --- | --- | --- | --- | --- | --- | --- | --- | --- | --- | --- | --- | --- | --- | --- |
| Patient No. | Age (years) | Gender | *S. pneumoniae* in blood culture | *S. pneumoniae* in sputum culture | *S. pneumoniae* in nasopharyngeal culture | Infecting serotype | Days between sample collection | Currently smoking | Comorbidity^a^ | SOFA score increase | Symptom duration before acute-phase sample | Acute-phase anti-CPS Ig level (AU) | Convalescent-phase anti-CPS Ig level (AU) | Plasma Spn9802 DNA concentration (copies/mL) |
| 1 | 54 | Female | Yes | Yes | Yes | 3 | 45 | No | No | 1 | 2 | 106 | 494 | - |
| 2 | 71 | Male | No | Yes | Yes | 3 | 27 | Yes | Yes | 1 | 1 | 150 | 116 | - |
| 3 | 57 | Female | Yes | Yes | Yes | 9V | 27 | No | No | 2 | 0 | 31 | 457 | - |
| 4 | 62 | Male | No | Yes | Yes | 9V | 30 | No | No | 2 | 8 | 83 | 115 | - |
| 5 | 43 | Male | No | Yes | Yes | 18C | 60 | Yes | No | 0 | 0 | 20 | 80 | 0 |
| 6 | 77 | Female | No | Yes | Yes | 23F | 28 | No | Yes | 2 | 5 | 54 | 81 | 0 |
| 7 | 59 | Female | No | Yes | No | 19A | 32 | No | No | 1 | 6 | 40 | 76 | 0 |
| 8 | 74 | Male | No | Yes | No | 3 | 25 | No | Yes | 3 | 2 | 37 | 87 | 1.8 *x* 10^2^ |
| 9 | 39 | Male | Yes | No | No | 7F | 81 | No | Yes | 3 | 3 | 12 | 525 | 0 |
| 10 | 31 | Male | Yes | No | Yes | 7F | 46 | No | No | 0 | 2 | 15 | 1230 | 0 |
| 11 | 59 | Male | No | No | Yes | 14 | 25 | Yes | Yes | 2 | 3 | 6 | 64 | - |
| 12 | 72 | Female | No | No | Yes | 19F | 28 | No | No | 0 | 0 | 26 | 410 | 0 |
| 13 | 31 | Male | No | No | Yes | 7F | 29 | No | No | 1 | 4 | 7 | 48 | 4.0 *x* 10^2^ |
| 14 | 37 | Male | No | Yes | Yes | 4 | 28 | Yes | No | 1 | 2 | 58 | 97 | - |
| 15 | 62 | Female | No | No | Yes | 7F | 28 | No | Yes | 4 | 0 | 56 | 207 | - |
| 16 | 67 | Female | Yes | No | Yes | 7F | 38 | No | No | 3 | 5 | 112 | 575 | - |
| 17 | 74 | Male | No | No | Yes | 3 | 33 | Yes | Yes | 2 | 1 | 60 | 77 | - |
| 18 | 90 | Male | No | Yes | Yes | 14 | 31 | No | Yes | 2 | 8 | 15 | 325 | 0 |
| 19 | 87 | Male | No | No | Yes | 3 | 31 | No | Yes | 5 | 0 | 80 | 61 | 0 |
| 20 | 40 | Male | No | Yes | Yes | 1 | 30 | Yes | No | 4 | 4 | 81 | 1754 | - |
| 21 | 41 | Male | No | Yes | No | 14 | 51 | Yes | No | 3 | 2 | 18 | 25 | - |
| 22 | 76 | Female | No | No | Yes | 14 | 31 | No | No | 5 | 8 | 35 | 25 | - |
| 23 | 60 | Female | No | Yes | Yes | 14 | 28 | No | No | 0 | 4 | 38 | 100 | - |
| 24 | 68 | Female | No | No | Yes | 14 | 81 | No | Yes | 0 | 21 | 65 | 144 | - |
| 25 | 89 | Female | Yes | No | Yes | 7F | 58 | No | Yes | 3 | 7 | 20 | 21 | 1.8 *x* 10^4^ |
| 26 | 84 | Female | No | No | Yes | 18C | 27 | No | Yes | 1 | 1 | 22 | 24 | 0 |
| 27 | 83 | Male | No | Yes | Yes | 4 | 38 | No | Yes | 2 | 1 | 76 | 197 | 0 |
| 28 | 89 | Male | No | Yes | Yes | 6B | 38 | No | No | 2 | 36 | 8 | 4 | - |
| 29 | 57 | Male | No | Yes | Yes | 3 | 25 | No | No | 1 | 0 | 114 | 109 | 0 |
| 30 | 46 | Female | No | Yes | Yes | 3 | 69 | Yes | No | 1 | 7 | 243 | 215 | 0 |
| 31 | 88 | Male | No | No | Yes | 9V | 33 | No | Yes | 4 | 1 | 102 | 372 | - |
| 32 | 72 | Female | No | No | Yes | 14 | 29 | Yes | Yes | 1 | 0 | 53 | 76 | - |
| 33 | 78 | Male | No | Yes | Yes | 19A | 33 | No | Yes | 2 | 3 | 24 | 21 | - |
| 34 | 77 | Male | No | Yes | Yes | 14 | 30 | No | Yes | 3 | 0 | 31 | 142 | 0 |
| 35 | 76 | Male | No | Yes | Yes | 14 | 61 | No | Yes | 3 | 1 | 117 | 39 | - |
| 36 | 61 | Female | Yes | Yes | Yes | 1 | 29 | Yes | Yes | 2 | 2 | 22 | 23 | - |
| 37 | 54 | Male | Yes | Yes | Yes | 3 | 31 | Yes | Yes | 2 | 4 | 81 | 172 | 4.5 *x* 10^3^ |
| 38 | 54 | Male | No | Yes | Yes | 3 | 26 | No | No | 3 | 6 | 62 | 81 | - |
| 39 | 58 | Male | No | No | Yes | 3 | 34 | Yes | No | 2 | 10 | 84 | 122 | 5.8 *x* 10^2^ |
| 40 | 79 | Female | Yes | No | Yes | 9V | 24 | No | No | 3 | 8 | 48 | 64 | 3.0 *x* 10^9^ |
| 41 | 54 | Female | No | No | Yes | 18C | 28 | No | No | 0 | 5 | 11 | 12 | - |
| 42 | 69 | Male | Yes | No | No | 19A | 31 | No | Yes | 4 | 3 | 27 | 2 | - |
| 43 | 85 | Female | Yes | No | No | 23F | 29 | No | Yes | 3 | 0 | 5 | 11 | 0 |
| 44 | 84 | Female | Yes | Yes | Yes | 23F | 82 | No | Yes | 1 | 4 | 16 | 14 | 0 |
| 45 | 89 | Male | No | Yes | Yes | 23F | 74 | No | Yes | 1 | 1 | 64 | 136 | - |
| 46 | 73 | Female | No | Yes | Yes | 23F | 26 | Yes | No | 2 | 11 | 101 | 118 | - |
| 47 | 85 | Male | Yes | No | No | 23F | 20 | No | No | 3 | 3 | 5 | 13 | - |
| 48 | 73 | Male | No | Yes | Yes | 19F | 30 | No | Yes | 3 | 3 | 96 | 162 | 0 |
| 49 | 83 | Female | Yes | No | Yes | 19F | 26 | No | No | 3 | 6 | 57 | 33 | 1.0 *x* 10^10^ |
| 50 | 62 | Female | No | Yes | No | 7F | 26 | Yes | Yes | 1 | 2 | 6 | 12 | - |
| 51 | 48 | Female | No | No | Yes | 3 | 22 | Yes | No | 1 | 6 | 62 | 68 | - |
| 52 | 91 | Female | Yes | Yes | Yes | 14 | 70 | No | No | 1 | 7 | 28 | 11 | - |
| 53 | 57 | Female | Yes | Yes | Yes | 7F | 31 | Yes | No | 2 | 5 | 73 | 12 | 0 |
| 54 | 23 | Male | No | No | Yes | 7F | 34 | No | No | 3 | 11 | 183 | 528 | 6.2 *x* 10^3^ |

^a^ One or more of any of the following diagnoses: chronic obstructive pulmonary disease, heart disease, diabetes mellitus, liver disease, renal insufficiency, neoplasm, or immunosuppression.
