# Supplementary material for: Corrected and Republished from: A Nonfunctional Opsonic Antibody Response Frequently Occurs after Pneumococcal Pneumonia and Is Associated with Invasive Disease
Source: mSphere. 2020 Dec 16;5(6):e01102-20. doi: 10.1128/mSphere.01102-20 (PMC7758726; doi:10.1128/mSphere.01102-20)
Supplement: TABLE S2 [file mSphere.01102-20-st002.docx]

| **Table S2.** | | | | |
| --- | --- | --- | --- | --- |
| Infecting serotype | Patient No. | Acute-phase serum OPA titer | Convalescent-phase serum OPA titer | Control serum OPA titer |
| 1 | 36 | 1 | 1 | 2,916 |
| 1 | 20 | 108 | 972 | 2,916 |
| 3 | 37 | 1 | 1 | 108 |
| 3 | 1 | 1 | 4 | 108 |
| 3 | 38 | 1 | 1 | 108 |
| 3 | 39 | 1 | 1 | 108 |
| 3 | 51 | 972 | 1 | 108 |
| 3 | 2 | 1 | 108 | 108 |
| 3 | 19 | 108 | 324 | 108 |
| 3 | 8 | 1 | 972 | 108 |
| 3 | 17 | 36 | 972 | 108 |
| 3 | 29 | 972 | 972 | 108 |
| 3 | 30 | 972 | 972 | 108 |
| 4 | 27 | 324 | 324 | 8,748 |
| 4 | 14 | 1 | 8,748 | 8,748 |
| 6B | 28 | 324 | 324 | 972 |
| 7F | 53 | 972 | 4 | 972 |
| 7F | 25 | 12 | 12 | 972 |
| 7F | 9 | 1 | 972 | 972 |
| 7F | 10 | 1 | 972 | 972 |
| 7F | 16 | 4 | 972 | 972 |
| 7F | 50 | 324 | 1 | 2,916 |
| 7F | 54 | 2,916 | 972 | 324 |
| 7F | 13 | 1 | 2,916 | 324 |
| 7F | 15 | 1 | 8,748 | 2,916 |
| 9V | 40 | 1 | 1 | 324 |
| 9V | 3 | 1 | 108 | 972 |
| 9V | 4 | 1 | 108 | 972 |
| 9V | 31 | 972 | 972 | 972 |
| 14 | 52 | 972 | 1 | 972 |
| 14 | 11 | 1 | 972 | 972 |
| 14 | 32 | 972 | 972 | 2,916 |
| 14 | 18 | 36 | 2,916 | 972 |
| 14 | 23 | 972 | 2,916 | 972 |
| 14 | 24 | 972 | 2,916 | 2,916 |
| 14 | 22 | 324 | 8,748 | 972 |
| 14 | 34 | 8,748 | 8,748 | 26,244 |
| 14 | 21 | 108 | 26,244 | 2,916 |
| 14 | 35 | 26,244 | 26,244 | 26,244 |
| 18C | 41 | 1 | 1 | 972 |
| 18C | 26 | 36 | 36 | 972 |
| 18C | 5 | 1 | 108 | 324 |
| 19A | 42 | 1 | 1 | 2,916 |
| 19A | 7 | 1 | 324 | 2,916 |
| 19A | 33 | 972 | 972 | 2,916 |
| 19F | 49 | 108 | 1 | 108 |
| 19F | 48 | 36 | 4 | 108 |
| 19F | 12 | 1 | 972 | 108 |
| 23F | 43 | 1 | 1 | 972 |
| 23F | 44 | 1 | 1 | 972 |
| 23F | 47 | 4 | 1 | 972 |
| 23F | 45 | 1 | 1 | 972 |
| 23F | 46 | 1 | 1 | 972 |
| 23F | 6 | 1 | 108 | 972 |
